# Systematic review of epidemiological studies on health effects associated with management of solid waste

**DOI:** 10.1186/1476-069X-8-60

**Published:** 2009-12-23

**Authors:** Daniela Porta, Simona Milani, Antonio I Lazzarino, Carlo A Perucci, Francesco Forastiere

**Affiliations:** 1 Department of Epidemiology, Regional Health Service Lazio Region, Rome, Italy; 2 Division of Epidemiology, Public Health and Primary Care, Imperial College, London, UK

## Abstract

**Background:**

Management of solid waste (mainly landfills and incineration) releases a number of toxic substances, most in small quantities and at extremely low levels. Because of the wide range of pollutants, the different pathways of exposure, long-term low-level exposure, and the potential for synergism among the pollutants, concerns remain about potential health effects but there are many uncertainties involved in the assessment. Our aim was to systematically review the available epidemiological literature on the health effects in the vicinity of landfills and incinerators and among workers at waste processing plants to derive usable excess risk estimates for health impact assessment.

**Methods:**

We examined the published, peer-reviewed literature addressing health effects of waste management between 1983 and 2008. For each paper, we examined the study design and assessed potential biases in the effect estimates. We evaluated the overall evidence and graded the associated uncertainties.

**Results:**

In most cases the overall evidence was inadequate to establish a relationship between a specific waste process and health effects; the evidence from occupational studies was not sufficient to make an overall assessment. For community studies, at least for some processes, there was limited evidence of a causal relationship and a few studies were selected for a quantitative evaluation. In particular, for populations living within two kilometres of landfills there was limited evidence of congenital anomalies and low birth weight with excess risk of 2 percent and 6 percent, respectively. The excess risk tended to be higher when sites dealing with toxic wastes were considered. For populations living within three kilometres of old incinerators, there was limited evidence of an increased risk of cancer, with an estimated excess risk of 3.5 percent. The confidence in the evaluation and in the estimated excess risk tended to be higher for specific cancer forms such as non-Hodgkin's lymphoma and soft tissue sarcoma than for other cancers.

**Conclusions:**

The studies we have reviewed suffer from many limitations due to poor exposure assessment, ecological level of analysis, and lack of information on relevant confounders. With a moderate level confidence, however, we have derived some effect estimates that could be used for health impact assessment of old landfill and incineration plants. The uncertainties surrounding these numbers should be considered carefully when health effects are estimated. It is clear that future research into the health risks of waste management needs to overcome current limitations.

## Introduction

"Waste management", that is the generation, collection, processing, transport, and disposal of solid waste is important for both environmental reasons and public health. There are a number of different options available for the management and treatment of waste including minimisation, recycling, composting, energy recovery and disposal. At present, an increasing amount of the resources contained in waste is recycled, but a large portion is incinerated or permanently lost in landfills. The various methods of waste management release a number of substances, most in small quantities and at extremely low levels. However, concerns remain about potential health effects associated with the main waste management technologies and there are many uncertainties involved in the assessment of health effects.

Several studies of the possible health effects on populations living in proximity of landfills and incinerators have been published and well-conducted reviews are available [1-4]. Both landfills and incinerators have been associated with some reproductive and cancer outcomes. However, the reviews indicate the weakness of the results of the available studies due to design issues, mainly related to a lack of exposure information, use of indirect surrogate measures, such as the distance from the source, and lack of control for potential confounders. As a result, there is great controversy over the possible health effects of waste management on the public due to differences in risk communication, risk perception and the conflicting interests of various stakeholders. Therefore, there is the need for an appropriate risk assessment that informs both policy makers and the public with the information currently available on the health risks associated with different waste management technologies. Of course, the current uncertainties should be taken into account.

Within the EU-funded INTARESE project [5], we aimed to assess potential exposures and health effects arising from solid wastes, from generation to disposal, or treatment. A key part in the health impact assessment was selecting or developing a suitable set of relative risks that link individual exposures with specific health endpoints. In this paper, we systematically reviewed the available epidemiological literature on health effects in the vicinity of landfills and incinerators and among workers at waste processing plants to derive usable excess risk estimates for health impact assessment. The degree of uncertainty associated with these estimates was considered.

## Methods

We considered epidemiological studies conducted on the general population with potential exposures from collecting, recycling, composting, incinerating, and landfilling solid waste. We also considered studies of employees of waste management plants as they may be exposed to the same potential hazards as the community residents, even if the intensity and duration of the exposure may differ. However, to limit our scope, we did not consider studies on biomarkers of exposure and health effects.

Relevant papers were found through computerized literature searches of MEDLINE and PubMed Databases from 1/1/1983 through 31/12/2008, using the MeSH terms "waste management" and "waste products" and the subheading "adverse effects". We identified 144 papers with this method. We also conducted a free search with several combinations of relevant key words (waste incinerator or landfill or composting or recycling) and (cancer or birth outcome or health effects), and 285 papers were identified. In addition, articles were traced through references listed in previous reviews [1-3,6-9], and in publications of the UK Department for Environment, Food and Rural Affairs [10]. Finally, we used information from two recent reviews of epidemiological studies on populations with potential exposures from toxic and hazardous wastes for reproductive [4], and cancer [11] outcomes, respectively.

The eligibility of all papers was evaluated independently by three observers, and disagreements were resolved by discussion. As indicated, studies on sewage treatment and on biological monitoring were not included. We also excluded articles in languages other than English, not journal articles, and six studies [12-17] conducted at the municipal level (usually small towns) where it was not possible to evaluate the extent of the population potentially involved and the possibility of exposure misclassification was high.

Papers were grouped according to the following criteria:

• waste management technologies: recycling, composting, incinerating, landfilling (considering controlled disposal of waste land and toxic or hazardous sites);

• health outcomes: cancers (stomach, colorectal, liver, larynx and lung cancer, soft tissue sarcoma, kidney and bladder cancer, non-Hodgkin's lymphoma, childhood cancer), birth outcomes (congenital malformations, low birth weight, multiple births, abnormal sex ratio of newborns), respiratory, skin and gastrointestinal symptoms or diseases.

We have reported in the appropriate tables (in the online additional files) for each paper: study design (e.g. geographical, cohort, cross-sectional, case-control study, etc.), population characteristics (subjects, country, age, sex), exposure measures (e.g. occupational exposure to waste incinerator by-products, residence near a landfill, etc.), and the main results (including control for major confounders) with respect to the quantification of the health effects studied. For each study we have evaluated the potential sources of uncertainty in the results due to design issues. In particular, the possibility that selection bias, information bias, or confounding could artificially increase or decrease the relative risk estimate has been noted in the tables using the plus/minus scale to indicate that effect estimates are likely to be overestimated (or underestimated) up to 20% (+/-), from 20 to 50% (++/--) and more than 50% (+++/---). Uncertainties were graded by two observers (SM and FF), who discussed the inconsistencies.

After a description of the available studies, the overall evaluation of the epidemiological evidence regarding the process/disease association was made based on the IARC (1999) criteria, and two categories were chosen, namely: "*Inadequate*" when the available studies were of insufficient quality, consistency, or statistical power to determine the presence or absence of a causal association; "*Limited*" when a positive association was observed between exposure and disease for which a causal interpretation is considered to be credible, but chance, bias, or confounding could not be ruled out with reasonable confidence. There were no instances where the category "sufficient" evidence could be used. Only when the specific process/disease association was judged as limited (suggestive evidence but not sufficient to infer causality) we decided to evaluate the strength of the association and to measure appropriate relative risks. For this purpose, we considered the set of studies providing the best evidence and assigned an overall level of scientific confidence of the specific effect estimate based on an arbitrary scale: very high, high, moderate, low, very low. This evaluation was made by three assessors (SM, DP, and FF).

## Results

A total of 49 papers were reviewed: 32 concerning health effects in communities in proximity to waste sites, and 17 on employees of waste management sites. The majority of community studies evaluated possible adverse health effects in relation to incinerators and landfills. We found little evidence on potential health problems resulting from environmental or occupational exposures from composting or recycling, and very little on storage/collection of solid waste. A description of the main findings follows.

## Studies of communities near landfills

One of the main problems in dealing with studies on landfill sites (an to some extent also for incinerators) is the distinction between sites for municipal solid wastes and sites for other wastes. The definition of different types of waste is far from being standardised across the world. The terms hazardous, special, toxic, industrial, commercial, etc, are variously applied in different countries and time periods to designate non-household wastes. In earlier time periods definitions were even less clear and some disposal sites may have switched categories (e.g. if they used to take industrial waste they may now only take municipal waste). Since two systematic reviews were already available for toxic wastes [4,11], we did not replicate the literature search, but summarized the evidence reported in the available reviews and tried to compare and discuss the results with studies where mainly municipal solid wastes were landfilled. The additional file 1 contain several details of the studies reviewed.

### Cancer

Russi et al. [11] carried out Medline searches of the peer-reviewed English language medical literature covering the period from January 1980 to June 2006 using the keywords "toxic sites" and "cancer", and identified articles from published reviews. They included 19 articles which fit the following selection criteria: 1) the study addressed either cancer incidence or cancer mortality as an endpoint, 2) the study was carried out in a community or a set of communities containing a known hazardous waste site; 3) the study had to address exposure from a specific waste site, rather than from a contaminated water supply resulted from multiple point sources. As the authors recognized, some of the location investigated included both toxic wastes and municipal solid wastes as in the study from Goldberg et al. [18] or Pukkala et al. [19]. There are two investigations considered in this review that are important to evaluate because of the originality of the approach (cohort study, [19] and due to the large size [20].

In Finland, Pukkala et al. [19] studied whether the exposure to landfills caused cancer or other chronic diseases in inhabitants of houses built on a former dumping area containing industrial and household wastes. After adjusting for age and sex, an excess number of male cancer cases were seen, especially for cancers of the pancreas and of the skin. The relative risk slightly increased with the number of years lived in the area. However, some uncertainties were likely to affect the results of the study with regards to the exposure assessment (-), outcome assessment (+) and presence of residual confounding (-).

Jarup et al. [20] examined cancer risks in populations living within 2 km of 9,565 (from a total of 19,196) landfill sites that were operational at some time from 1982 to 1997 in Great Britain. No excess risks of cancers of the bladder and brain, hepato-biliary cancer or leukaemia were found, after adjusting for age, sex, calendar year and deprivation. The study was very large and had high power, however misclassification of exposure could have decreased the possibility of detecting an effect (--).

Based on the findings and on the evaluation of the quality of the studies, Russi et al. [11] concluded that epidemiological studies of populations living in the vicinity of a toxic waste site have not produced evidence of adequate quality to establish a casual link between toxic waste exposures and cancer risk. In our terms, the evidence may be considered as "inadequate".

In addition to the articles reviewed by Russi et al. [11], we reviewed the article by Michelozzi et al. [21], which investigated the mortality risk in a small area of Italy (Malagrotta, Rome) with multiple sources of air contamination (a very large waste disposal site serving the entire city of Rome, a waste incinerator plant, and an oil refinery plant). Standardised Mortality Ratios (SMRs) were computed in bands of increasing distance from the plants, up to a radius of 10 km. No association was found between proximity to the sites and cancer of various organs, in particular liver, lung, and lymph haematopoietic cancer, however, mortality from laryngeal cancer declined with distance from the pollution sources, and a statistically significant trend remained after adjusting for a four-level index of socio-economic status. The main uncertainty of the study is related to the exposure assessment (--) since only distance was considered thus decreasing the possibility of detecting an effect. There are also uncertainties in using mortality to estimate cancer incidence in proximity to a suspected source of pollution (+). On the other hand, even though the authors did adjust for an area-based index of deprivation, residual confounding (+) from socioeconomic status was likely.

In summary, there is inadequate evidence of an increased risk of cancer for communities in proximity of landfills. The three slightly positive studies from Goldberg et al. [18], Pukkala et al. [19] and Michelozzi et al. [21] are not consistent.

### Birth defects and reproductive disorders

Saunders [4] reviewed 29 papers examining the relationship between residential proximity to landfill sites and the risk of an adverse birth outcome. The review included either studies on municipal waste or on hazardous waste. Eighteen papers reported some significant association between adverse reproductive outcome and residence near a landfill site. Two of the strongest papers conducted on hazardous waste landfill sites in Europe (EUROHAZCON) found similarly moderate but significant associations between residential proximity (within 3 km) to hazardous waste sites and both chromosomal [22] (Odds Ratio, OR: 1.41, 95%CI: 1.00-1.99) and non-chromosomal [23] (OR: 1.33, 95%CI: 1.11-1.59) congenital anomalies.

Included in the Saunders's review [4] is the national geographical comparison study on landfills in the UK by Elliott et al. [24]. This study investigated the risk of adverse birth outcomes in populations living within two km of 9,565 landfill sites in Great Britain, operational at some time between 1982 and 1997, compared with those living further away (reference population). The sites included 774 sites for special (hazardous) waste, 7803 for non-special waste and 988 handling unknown waste; a two km zone was defined around each site to detect the likely limit of dispersion for landfill emissions, including 55% of the national population. Among the 8.2 million live births and 43,471 stillbirths, 124,597 congenital anomalies (including miscarriage) that were examined, there were: neural tube defects, cardiovascular defects, abdominal wall defects, hypospadias and epispadias, surgical correction of gastroschisis and exomphalos; low and very low birth weights were also found , defined as less than 2500 g and less than 1500 g, respectively. The main analysis, conducted for all landfill sites during their operation and after closure, found a small, but still statistically significant, increased risk of total and specific anomalies (OR: 1.01, 95%CI: 1.005-1.023) in populations living within 2 Km, and also an increased risk of low (OR: 1.05, 95%CI: 1.047-1.055) and very low birth weight (OR: 1.04, 95%CI: 1.03-1.05). Additional analyses were carried out separately for sites handling special waste and non-special waste, and in the period before and after opening, for the 5,260 landfills with available data. After adjusting for deprivation and other potential confounding variables (sex, year of birth, administrative region), there was a small increase in the relative risks for low and very low birth weight and for all congenital anomalies, except for cardiovascular defects. The risks of all congenital anomalies were higher for people living near special waste disposals (OR: 1.07 CI95%:1.04-1.09) compared to non-special waste disposals (OR: 1.02, CI95%:1.01-1.03). There was no excess risk of stillbirth. On these bases, the author [4] concluded that while most studies reporting a positive association are of good quality, over half report no association with any adverse birth outcome and most of the latter are also well conducted. The review considered that the evidence of an association of residence near a landfill with adverse birth outcomes as unconvincing.

After the review by Saunders [4], we considered four additional studies examining reproductive effects of landfill emissions.

Elliot et al. recently updated the previous study [25] in order to evaluate whether geographical density of landfill sites was related to congenital anomalies. The analysis was restricted to 8804 sites operational at some time between 1982 and 1997. There were 607 sites handling special (hazardous) waste and 8197 handling non-special or unknown waste type. The exposure assessment took into account the overlap of the two km buffers around each site, to define an index of exposure with four levels of increasing landfill density. Several anomalies (hypospadias and epispadias, cardiovascular defects, neural tube defects and abdominal wall defects) were evaluated. The analysis was carried out separately for special and non-special waste sites and was adjusted for deprivation, presence or absence of a local congenital anomalies register and maternal age. The study found a weak association between intensity of hazardous sites and some congenital anomalies (all, cardiovascular, hypospadia and epispadias).

The studies conducted in the United Kingdom suffer from the same limitations, namely the possibility that misclassification of exposure could have decreased the relative risk estimates to some extent (--); on the other hand, there are several uncertainties related to the quality of reporting and registration of congenital malformations. In the latter case, a positive bias is more likely (++). For the recent report by Elliott et al. [25], location uncertainties and differential data reliability regarding the sites, together with the use of distance as the basis for exposure classification, limit the interpretation of the findings (--).

In Denmark, Kloppenborg et al. [26] marked the geographical location of 48 landfills and used maternal residence as the exposure indicator in a study of congenital malformations. The authors found no association between landfill location and all congenital anomalies or of the nervous system, and a small excess risk for congenital anomalies of the cardiovascular system. Potential confounding from socioeconomic status is the major limitation of this study (+++).

Jarup et al. [27] studied the risk of Down's syndrome in the population living near 6829 landfills in England and Wales. People were considered exposed if they lived in a two-km zone around each site, people beyond this zone were the reference group. A two-year lag period between potential exposure of the mother and her giving birth to a Down's syndrome child was allowed. The analysis was adjusted for maternal age, urban-rural status and deprivation index. No statistically significant excess risk was found in the exposed populations, regardless of waste type.

Finally, Gilbreath et al. [28] studied births in 197 Native Alaskan villages containing open dumpsites with hazardous waste, scoring the exposure into high, intermediate and low hazard level on the basis of maternal residence. The authors found an association between higher levels of hazard and low birth weight and intrauterine growth retardation. The major limit of the study is the low specificity of the exposure definition.

In summary, an increased risk of congenital malformations and of low birth weight has been reported from studies conducted in the UK. When compared with the results from studies conducted in proximity of hazardous waste sites, studies in proximity of non-toxic waste landfills provide lower effect estimates. The main uncertainty of these studies is the completeness of data on birth defects, the use of distance from the sites for exposure classification, and the classification as toxic and non-toxic waste sites.

### Respiratory diseases

A study conducted by Pukkala et al. [19] in Finland evaluated prevalence of asthma in relation to residence in houses built on a former dumping area containing industrial and household wastes. Prevalence of asthma was significantly higher in the dump cohort than in the reference cohort (living nearby but outside the landfill site). Unfortunately, this study has not been replicated and the overall evidence may be considered inadequate.

## Studies of landfills workers

Only one study on landfill workers was reviewed. Gelberg et al. [29] conducted a cross-sectional study to examine acute health effects among employees working for the New York City Department of Sanitation, focusing on Fresh Kills landfill employees. Telephone interviews conducted with 238 on-site and 262 off-site male employees asked about potential exposures both at home and work, health symptoms for the previous six months, and other information (social and recreational habits, socio-economic status). Landfill workers reported a significantly higher prevalence of work-related respiratory, dermatological, neurologic and hearing problems than controls. Respiratory and dermatologic symptoms were not associated with any specific occupational title or task, other than working at the landfill, and the association remained, even after controlling for smoking status.

## Studies of communities living near incinerators

Twenty-one epidemiologic studies conducted on residents of communities with solid waste incinerators have been reviewed and their characteristics are listed in the additional file 2.

### Cancer

Eleven studies have been reviewed on cancer risk in relation with incinerators, usually old plants with high polluting characteristics. The studies are reported below by country.

In the United Kingdom, Elliott et al. [30] investigated cancer incidence between 1974 and 1987 among over 14 million people living near 72 solid waste incinerator plants. Data on cancer incidence among the residents, obtained from the national cancer registration programme, were compared with national cancer rates, and numbers of observed and expected cases were calculated after stratifying for deprivation, based on the 1981 census. Observed-expected ratios were tested for decline in risk up to 7.5 km away. The study was conducted in two stages: the first involved a stratified random sample of 20 incinerators and, based on the findings, a number of cancers were then further studied around the remaining 52 incinerators (second stage). Over the two stages of the study there was a statistically significant (*p *< 0.05) decline in risk with distance from incinerators for all cancers, stomach, colorectal, liver and lung cancer. The use of distance as the exposure variable in this study could have led to some degree of misclassification (--). On the other hand, the same authors observed that residual confounding (+) as well as misdiagnosis (+) might have increased the risk estimates. When further analyses were made, including a histological review of liver cancer cases [31], the risk estimates were lower (0.53-0.78 excess cases per 10^5 ^per year within 1 km, instead of 0.95 excess cases per 10^5 ^as previously estimated).

Using data on municipal solid waste incinerators from the initial study by Elliott et al. [30], Knox [32] examined a possible association between childhood cancers and industrial emissions, including those from incinerators. From a database of 22,458 cancer deaths that occurred in children before their 16^th ^birthday between 1953 and 1980, he extracted 9,224 cases known to have moved at least 0.1 km in their life time, and using a newly developed technique of analysis, he compared distances from the suspected sources to the birth addresses and to the death addresses. The childhood-cancer/leukaemia data showed highly significant excesses of moves away from birthplaces close to municipal incinerators, but the specific effects of the municipal incinerators could not be separated clearly from those of nearby industrial sources of combustion. Misclassification of exposure is the main limit of this paper (--).

In France, Viel et al. [33] detected a cluster of patients with non-Hodgkin's lymphoma (NHL) and soft tissue sarcoma around a French municipal solid waste incinerator with high dioxin emissions. To better explore the environmental origin of the cluster suggested by these findings, Floret et al. [34] carried out a population-based case-control study in the same area, comparing 222 incident cases of NHL diagnosed between 1980 and 1995 and controls randomly selected from the 1990 census. The risk of developing lymphomas was 2.3 times higher among individuals living in the area with the highest dioxin concentration than among those in the area with the lowest concentration. Given that a model was used to attribute exposure to cases and controls, a random misclassification could have reduced the effect estimates (--). Based of these results, a nationwide study on NHL was conducted [35]. A total of 13 incinerators in France were investigated and dispersion modelling was used to estimate ground-level dioxin concentration. Information about the exposure levels and potential confounders was available at the census block level. A positive association between dioxin level and NHL was found with a stronger effect among females. Although the study represents an improvement regarding exposure assessment compared to investigations based on distance from the source, it should be noted that the analysis was conducted at the census block level and the possibility of misclassification of the exposure (-) as well as of residual confounding from socioeconomic status (+) remains.

Viel et al. [36] have recently reported the findings from a case-control study on breast cancer. There was no association or even a negative association between exposure to dioxin and breast cancer in women younger or older than 60 years, respectively, living near a French municipal solid waste incinerator with high exposure to dioxin. Design issues and residual confounding from age and other factors (---) limit the interpretations of the study.

In Italy, Biggeri et al. [37] conducted a case-control study in Trieste to investigate the relationship between multiple sources of environmental pollution and lung cancer. Based on distance from the sources, spatial models were used to evaluate the risk gradients and the directional effects separately for each source, after adjusting for age, smoking habits, likelihood of exposure to occupational carcinogens, and levels of air particulate. The results showed that the risk of lung cancer was inversely related to the distance from the incinerator, with a high excess relative risk very near the source and a very steep decrease moving away from it. The main problem of the study is the difficulty to separate the effects of other sources of pollution based on distance, and the possibility of potential confounding from other sources remains (++). An excess risk of lung cancer was also found in females living in two areas of the province of La Spezia (Italy) exposed to environmental pollution emitted by multiple sources, including an industrial waste incinerator [38]. Again in this study the limited exposure assessment could have decreased the risk estimates (--), but positive confounding from other sources is very likely.

A case-control study by Comba et al. [39] showed a significant increase in risk of soft tissue sarcomas associated with residence within two km of an industrial waste incinerator in the city of Mantua, with a rapid decrease in risk at greater distances. There is a slight likelihood that increased attention to the diagnosis for this form of cancer in the vicinity of the plant could have introduced a small bias (+) in the risk estimate. Another case-control study, carried out in the province of Venice by Zambon et al. [40] analyzed the association between soft-tissue sarcoma and exposure to dioxin in a large area with 10 municipal solid waste incinerators. The authors found a statistically significant increase in the risk of sarcoma in relation to both the level and the length of environmental modelled exposure to dioxin-like substances. The results were more significant for women than for men.

In summary, although several uncertainties limit the overall interpretation of the findings, there is limited evidence that people living in proximity of an incinerator have increased risk of all cancers, stomach, colon, liver, lung cancers based on the studies of Elliott et al. [30]. Specific studies on incinerators in France and in Italy suggest an increased risk for non-Hodgkin's lymphoma, and soft-tissue sarcoma.

### Birth defects and reproductive disorders

Six studies examined reproductive effects of incinerator emissions (see additional file 2).

Jansson et al. [41] analysed whether the incidence of cleft lip and palate in Sweden increased since operation of a refuse incineration plant began. The results of this register study, based on information from the central register of malformations and the medical birth register, did not demonstrate an increased risk.

A study by Lloyd et al. [42] examined the incidence of twin births between 1975 and 1983 in two areas near a chemical and a municipal waste incinerator in Scotland: after adjusting for maternal age, an increased frequency of twinning in areas exposed to air pollution from incinerators was seen. In the same study areas, Williams et al. [43] investigated gender ratios, at various levels of geographical detail and using three-dimensional mapping techniques: analyses in the residential areas at risk from airborne pollution from incinerators showed locations with statistically significant excesses of female births.

To investigate the risk of stillbirth, neonatal death, and lethal congenital anomaly among infants of mothers living close to incinerators (and crematoriums), Dummer et al. [44] conducted a geographical study in Cumbria (Great Britain). After adjusting for social class, year of birth, birth order, and multiple births, there was an increased risk of lethal congenital anomaly, in particular spina bifida and heart defects.

Subsequently, Cordier et al. [45] studied communities with fewer than 50,000 inhabitants surrounding the 70 incinerators that operated for at least one year from 1988 to 1997 in France. Each exposed community was assigned an exposure index based on a Gaussian plume model, estimating concentrations of pollutants per number of years the plant had operated. The results were adjusted for year of birth, maternal age, department of birth, population density, average family income, and when available, local road traffic. The rate of congenital anomalies was not significantly higher in exposed compared with unexposed communities; only some subgroups of congenital anomalies, specifically facial cleft and renal dysplasia, were more frequent in the exposed communities.

Tango et al. [46] investigated the association of adverse reproductive outcomes with mothers living within 10 km of 63 municipal solid waste incinerators with high dioxin emission levels (above 80 ng international toxic equivalents TEQ/m^3^) in Japan. To calculate the expected number of cases, national rates based on all live births, fetal deaths and infant deaths occurred in the study area during 1997-1998 were used and stratified by potential confounding factors available from the corresponding vital statistics records: maternal age, gestational age, birth weight, total previous deliveries, past experience of fetal deaths, and type of paternal occupation. None of the reproductive outcomes studied showed statistically significant excess within two km of the incinerators, but a statistically significant decline in risk with distance from the incinerators was found for infant deaths and for infant deaths with congenital anomalies, probably due to dioxin emissions from the plants.

In sum, there are multiple reports of increased risk of congenital malformations among people living close to incinerators but there are no consistencies between the investigated outcomes. The overall evidence may be considered as limited. The study by Cordier et al. [45] provides the basis for risk quantifications at least for facial cleft and renal dysplasia. Quantification for other reproductive disorders is more difficult.

### Respiratory and skin diseases or symptoms

Four studies examined respiratory and/or dermatologic effects of incinerator emissions (see additional file 2).

Hsiue et al. [47] evaluated the effect of long-term air pollution resulting from wire reclamation incineration on respiratory health in children. 382 primary school children who resided in one control and three polluted areas in Taiwan were chosen for this study. The results revealed a decrement in pulmonary function (including forced vital capacity and forced expiratory volume in one second) of those residents in the vicinity of incineration sites.

Shy et al. [48] studied the residents of three communities having, respectively, a biomedical and a municipal incinerator, and a liquid hazardous waste-burning industrial furnace, and then compared results with three matched-comparison communities. After adjustment for several confounders (age, sex, race, education, respiratory disease risk factors), no consistent differences in the prevalence of chronic or acute respiratory symptoms resulted between incinerator and comparison communities. Additionally, no changes in pulmonary function between subjects of an incinerator community and those of its comparison community resulted from the study by Lee et al. [49], based on a longitudinal component from the Health and Clean Air study by Shy et al. [48].

Miyake et al. [50] examined the relationship between the prevalence of allergic disorders and general symptoms in Japanese children and the distance of schools from incineration plants, measured using geographical information systems. After adjusting for grade, socio-economic status and access to health care per municipality, schools closer to the nearest municipal waste incineration plant were associated with an increased prevalence of wheeze and headache; there was no evident relationship between the distance of schools from such plants and the prevalence of atopic dermatitis. The main factors that may have affected the relative risk estimates in this study could be reporting bias (++) and residual confounding from socioeconomic status (++).

In sum, although the intensive study conducted by Shy et al. [48] did not show respiratory effects, there are some indications of an increased risk of respiratory diseases, especially in children. However, the uncertainty related to outcome assessment and residual confounding is very high and the overall evidence may be considered inadequate.

## Occupational studies on incinerator employees

Four studies conducted on incinerator employees were reviewed (see additional file 3).

In 1997, Rapiti et al. [51] conducted a retrospective mortality study on 532 male workers employed at two municipal waste incinerators in Rome (Italy) between 1962 and 1992. Standardized mortality ratios (SMRs) were computed using regional population mortality rates. Mortality from all causes resulted significantly lower than expected, and all cancer mortality was comparable with that of the general population. Mortality from lung cancer was lower than expected, but an increased risk was found for stomach cancer: analysis by latency since first exposure indicated that this excess risk was confined to the category of workers with more than 10 years since first exposure.

Bresnitz et al. [52] studied 89 of 105 male incinerator workers in Philadelphia, employed at the time of the study in late June 1988. Based on a work site analysis, workers were divided into potentially high and low exposure groups, and no statistically significant differences in pulmonary function were found between the two groups, after adjusting for smoking status.

A similar study was conducted by Hours et al. [53]: they analysed 102 male workers employed by three French urban incinerators during 1996, matched for age with 94 male workers from other industrial activities. The exposed workers were distributed into 3 exposure categories based on air sampling at the workplace: crane and equipment operators, furnace workers, and maintenance and effluent-treatment workers. An excess of respiratory problems, mainly daily cough, was more often found in the exposed groups, and a significant relationship between exposure and decreases in several pulmonary parameters was also observed, after adjusting for tobacco consumption and centre. The maintenance and effluent group, and the furnace group had elevated relative risks for skin symptoms.

In the same year, Takata et al. [54] conducted a cross-sectional study in Japan on 92 workers from a municipal solid waste incinerator to investigate the health effects of chronic exposure to dioxins. The concentrations of these chemicals among the blood of the workers who had engaged in maintenance of the furnace, electric dust collection, and the wet scrubber of the incinerator were higher compared with those of residents in surrounding areas, but there were no clinical signs or findings correlated to blood levels of dioxins.

In sum, there are some studies that suggest increased gastric cancer and respiratory problems among incinerators workers. However, there are a great number of uncertainties, which make it difficult to derive conclusions.

## Epidemiological studies of health effects of other waste management processes

Twelve epidemiologic studies on the potential adverse health effects of other waste management practices are reviewed and listed in additional file 4.

### Waste collection

Ivens et al. [55] investigated the adverse health effects among waste collectors in Denmark. In a questionnaire-based survey among 2303 waste collectors and a comparison group of 1430 male municipal workers, information on self-reported health status and working conditions was collected and related to estimated bioaerosol exposure. After adjusting for several confounders (average alcohol consumption per day, smoking status, and the psychosocial exposure measures support/demand ), a dose-response relationship between level of exposure to fungal spores and self-reported diarrhoea was indicated, meaning that the higher the weekly dose, the more reports of gastrointestinal symptoms.

In contrast with these results, a study of 853 workers employed by 27 municipal household waste collection departments in Taiwan did not find an excess of gastrointestinal symptoms [56]. The workers answered a questionnaire and were classified into two occupational groups by specific exposures based on the reported designation of their specific task. The exposed group included those working in the collection of mixed domestic waste, front runner or loader, collection of separated waste and special kinds of domestic waste (paper, glass, etc.), garden waste, bulky waste for incineration, and the vehicle driver; the control group included accountants, timekeepers, canteen staff, personnel, and other office workers. No significant differences were found in the prevalence of gastrointestinal symptoms, but results indicated that all respiratory symptom prevalence, except dyspnoea, were significantly higher in the exposed group, after adjusting for age, gender, education, smoking status, and duration of employment.

### Composting facilities

In a German cross sectional study by Bünger et al. [57], work related health complaints and diseases of 58 compost workers and 53 bio-waste collectors were investigated and compared with 40 control subjects. Compost workers had significantly more symptoms and diseases of the skin and the airways than the control subjects. No correction was performed for the confounding effect of smoking, as there were no significant differences in the smoking habits of the three groups.

A subsequent study in Germany by Herr et al. [58] examined the health effects on community residents of bio-aerosol, emitted by a composting plant. A total of 356 questionnaires from residents living at different distances from the composting site, and from unexposed controls were collected: self-reported prevalence of health complaints over past years, doctors' diagnoses, as was residential odor annoyance; microbiological pollution was measured simultaneously in residential outdoor air. Reports of airway irritation were associated with residency in the highest bio-aerosol exposure category, 150-200 m (versus residency >400-500 m) from the site, and periods of residency more than five years.

Bünger et al. [59] conducted a prospective cohort study to investigate, in 41 plants in Germany, the health risks of compost workers due to long term exposure to organic dust that specifically focused on respiratory disorders. Employees, exposed and not exposed to organic dust, were interviewed about respiratory symptoms and diseases in the last 12 months and had a spirometry after a 5-year follow-up. Exposure assessment was conducted at 6 out of 41 composting plants and at the individual level. Eyes, airways and skin symptoms were higher in compost workers than in the control group. There was also a steeper decline of Forced Vital Capacity among compost workers compared to control subjects, also when smoking was considered.

### Materials recycling facilities

There are no epidemiological studies of populations living near materials recycling facilities; only studies on employees are available.

In the already-quoted study by Rapiti et al. [51] on workers at two municipal plants for incinerating and garbage recycling, increased risk was found for stomach cancer in employees who had worked there for at least 10 years, while lung cancer mortality risk was lower than expected.

In the study by Rix et al. [60], 5377 employees of five paper recycling plants in Denmark between 1965 and 1990 were included in a historical cohort, and the expected number of cancer cases was calculated from national rates. The incidence of lung cancer was slightly higher among men in production and moderately higher in short term workers with less than 1 year of employment; there was significantly more pharyngeal cancer among males, but this may have been influenced by confounders such as smoking and alcohol intake.

Sigsgaard et al. [61] conducted a cross-sectional study to examine the effect of shift changes on lung function among 99 recycling workers (resource recovery and paper mill workers), and correlated these findings with measurements of total dust and endotoxins. Exposure to organic dust caused a fall in FEV_1 _over the work shift, and this was significantly associated with exposure to organic dust; no significant association was found between endotoxin exposure and lung function decreases.

The same authors [62] also analysed skin and gastrointestinal symptoms among 40 garbage handlers, 8 composters and 20 paper sorters from all over Denmark, and found that garbage handlers had an increased risk of skin itching, and vomiting or diarrhoea.

In a nationwide study, Ivens et al. [63] reported findings of self-reported gastrointestinal symptoms by self-reported type of plant. A questionnaire based survey among Danish waste recycling workers at all composting, biogas-producing, and sorting plants collected data on occupational exposures (including questions on type of plant, type of waste), present and past work environment, the psychosocial work environment, and health status. Prevalence rate ratios adjusted for other possible types of job and relevant confounders were estimated with a comparison group of non-exposed workers, and an association was found between sorting paper and diarrhoea, between nausea and work at plastic sorting plants, and non-significantly between diarrhoea and work at composting plants.

The health status of workers employed in the paper recycling industry was also studied by Zuskin et al. [64]. A group of 101 male paper-recycling workers employed by one paper processing plant in Croatia, and a group of 87 non-exposed workers employed in the food packing industry was studied for the prevalence of chronic respiratory symptoms, and results indicated significantly higher prevalence of all chronic respiratory symptoms were found in paper workers compared with controls.

Gladding et al. [65] studied 159 workers from nine materials recovery facilities (MRFs) in the United Kingdom. Total airborne dust, endotoxins, (1-3)-beta-D-glucan were measured, and a questionnaire-survey was completed. The results suggest that materials recovery facilities workers exposed to higher levels of endotoxins and (1-3)-beta-D-glucan at their work sites experience various work-related symptoms, and that the longer a worker is in the MRF environment, the more likely he is to become affected by various respiratory and gastrointestinal symptoms.

## Choosing relative risk estimates for health impact assessment of residence near landfills and incinerators

The reviewed studies have been used to summarize the evidence available, as indicated in table 1. When the overall degree of evidence was considered "inadequate" we decided not to propose a quantitative evaluation of the relative risk; when we arrived to a conclusion that "limited" evidence was available, relative risk estimates were extracted for use in the health impact assessment process. Table 2 summarizes the relevant and reliable figures for health effects related to landfills and incinerators. For each relative risk the distance from the source has been reported as well as the overall level of confidence of the effect estimates based on an arbitrary scale: very high, high, moderate, low, very low.

**Table 1:** Summary of the overall epidemiologic evidence on municipal solid waste disposal: landfills and incinerators.

HEALTH EFFECT	LEVEL OF EVIDENCE	
	**LANDFILLS**	**INCINERATORS**
All cancer	Inadequate	Limited
Stomach cancer	Inadequate	Limited
Colorectal cancer	Inadequate	Limited
Liver cancer	Inadequate	Limited
Larynx cancer	Inadequate	Inadequate
Lung cancer	Inadequate	Limited
Soft tissue sarcoma	Inadequate	Limited
Kidney cancer	Inadequate	Inadequate
Bladder cancer	Inadequate	Inadequate
Non Hodgkin's lymphoma	Inadequate	Limited
Childhood cancer	Inadequate	Inadequate
Total birth defects	Limited	Inadequate
Neural tube defects	Limited	Inadequate
Orofacial birth defects	Inadequate	Limited
Genitourinary birth defects	Limited*	Limited**
Abdominal wall defects	Inadequate	Inadequate
Gastrointestinal birth defects§	Inadequate	Inadequate
Low birth weight	Limited	Inadequate
Respiratory diseases or symptoms	Inadequate	Inadequate

"*Inadequate*": available studies are of insufficient quality, consistency, or statistical power to decide the presence or absence of a causal association. "*Limited*": a positive association has been observed between exposure and disease for which a causal interpretation is considered to be credible, but chance, bias, or confounding could not be ruled out with reasonable confidence.

* Hypospadias and epispadias

** Renal dysplasia

§ The original estimates were given for "surgical corrections of gastroschisis and exomphalos"

**Table 2:** Relative risk estimates for community exposure to landfills and incinerators

Health effect	Distance from the source	Relative Risk (Confidence Interval)	Level of confidence**
**Landfills**			
***Congenital malformations ***[24]			
All congenital malformations	Within 2 km	1.02 (99% CI = 1.01-1.03)	Moderate
Neural tube defects	Within 2 km	1.06 (99% CI = 1.01-1.12)	Moderate
Hypospadias and epispadias	Within 2 km	1.07 (99% CI = 1.04-1.11)	Moderate
Abdominal wall defects	Within 2 km	1.05 (99% CI = 0.94-1.16)	Moderate
Gastroschisis and exomphalos*	Within 2 km	1.18 (99% CI = 1.03-1.34)	Moderate
***Low birth weight ***[24]	Within 2 km	1.06 (99% CI = 1.052-1.062)	High
Very low birth weight	Within 2 km	1.04 (99% CI = 1.03-1.06)	High
**Incinerators**			
***Congenital malformations ***[45]			
Facial cleft	Within 10 km	1.30 (95% CI = 1.06-1.59)	Moderate
Renal dysplasia	Within 10 km	1.55 (95% CI = 1.10-2.20)	Moderate
***Cancer ***[30]			
All cancer	Within 3 km	1.035 (95% CI = 1.03-1.04)	Moderate
Stomach cancer	Within 3 km	1.07 (95% CI = 1.02-1.13)	Moderate
Colorectal cancer	Within 3 km	1.11 (95% CI = 1.07-1.15)	Moderate
Liver cancer	Within 3 km	1.29 (95% CI = 1.10-1.51)	High
Lung cancer	Within 3 km	1.14 (95% CI = 1.11-1.17)	Moderate
Soft-tissue sarcoma	Within 3 km	1.16 (95% CI = 0.96-1.41)	High
Non-Hodgkin's lymphoma	Within 3 km	1.11 (95% CI = 1.04-1.19)	High

*The original estimates were given for "surgical corrections of..". **The following scale for the level of confidence has been adopted: very high, high, moderate, low, very low.

### Landfills

From the review presented above and following the work already made by Russi et al. [11], it is clear that the studies on cancer are not sufficient to draw conclusions regarding health effects near landfills, both with toxic and non-toxic wastes. The largest study conducted in England by Jarup et al. [21] does not suggest an increase in the cancer types that were investigated. Investigations of other chronic diseases are lacking, especially of respiratory diseases, yet there is one indication of an increased risk of asthma in adults [19], but with no replication of the findings. Overall, the evidence that living near landfills may be associated with health effects in adults is inadequate.

A slightly different picture appears for congenital malformations and low birth weight, where limited evidence exists of an increased risk for infants born to mothers living near landfill sites. The relevant results come from the European EUROHAZCON Study [23] and the national investigation from Elliott et al. [24]. In the UK report, statistically significant higher risk were found for all congenital malformations, neural tube defects, abdominal wall defects, surgical correction of gastroschisis and exomphalos, and low and very low birth weight for births to people living within two km of the sites, both of hazardous and non-hazardous waste. Although several alternative explanations, including ascertainment bias, and residual confounding cannot be excluded in the study, Elliott et al. [24] provide quantitative effect estimates whose level of confidence can be considered as moderate.

### Incinerators

Quantitative estimates of excess risk of specific cancers in populations living near solid waste incinerator plants were provided by Elliott et al. [30]. We have reported in table 2 the effect estimates for all cancers, stomach, colon, liver, and lung cancer based on their "second stage" analysis. There was an indication of residual confounding from socioeconomic status near the incinerators and a concern of misdiagnosis among registrations and death certificates for liver cancer. The histology of the liver cancer cases was reviewed, re-estimating the previously calculated excess risk (from 0.95 excess cases 10^-5^/year to between 0.53 and 0.78 excess cases 10^-5^/year). We then graded the confidence of the assessment for these tumours as "moderate" with the exception of liver cancer (high) since the misdiagnosis was reassessed and the extent of residual confounding was lower. In the study by Elliott et al. [30] no significant decline in risk with distance for non-Hodgkin's lymphoma and soft tissue sarcoma was found. However, the studies of Viel et al. [33] and Floret et al. [34] conducted in France and the studies from Comba et al. [39] and Zambon et al. [40] in Italy provide some indications that an excess of these forms of cancers may be related to emissions of dioxins from incinerators. As a result, we provided effect estimates in table 2 also for non-Hodgkin's lymphoma and soft tissue sarcoma as derived from the conservative "first stage" analysis conducted by Elliott et al. [30]. We graded the level of confidence of these relative risk estimates as "high".

With regards to congenital malformations near incinerators, Cordier et al. [45] provided effect estimates for facial cleft and renal dysplasia, as they were more frequent in the "exposed" communities living within 10 km of the sites. Other reproductive effects, such as an effect on twinning rates or gender determination, have been described; however the results are inadequate.

## Conclusions

We have conducted a systematic review of the literature regarding the health effects of waste management. After the extensive review, in many cases the overall evidence was inadequate to establish a relationship between a specific waste process and health effects. However, at least for some associations, a limited amount of evidence has been found and a few studies were selected for a quantitative evaluation of the health effects. These relative risks could be used to assess health impact, considering that the level of confidence in these effect estimates is at least moderate for most of them.

Most of the reviewed studies suffer from limitations related to poor exposure assessment, aggregate level of analysis, and lack of information on relevant confounders. It is clear that future research into the health risks of waste management requires a more accurate characterization of individual exposure, improved knowledge of chemical and toxicological data on specific compounds, multi-site studies on large populations to increase statistical power, approaches based on individuals rather than communities and better control of confounding factors.

## List of abbreviations used

EU: European Union; INTARESE: Integrated Assessment of Health Risks of Environmental Stressors in Europe; NHL: non-Hodgkin's Lymphoma; OR: Odds ratio; TEQ: Toxic Equivalent.

## Competing interests

The authors declare that they have no competing interests.

## Authors' contributions

DP participated in the design of the study, conducted the systematic review and drafted the manuscript. SM conducted the systematic review and contributed to draft the manuscript. AIL participated in the systematic review and contributed to draft the manuscript. CAP helped to conceive of the study and to write and revise the manuscript. FF conceived and coordinated the study and helped to write and revise the manuscript. All authors have read and approved the final manuscript.

## Supplementary Material

Additional file 1 **Studies on landfills**. The data provided represent a brief description of the studies on populations living near landfills. Click here for file

Additional file 2 **Studies on incinerators**. The data provided represent a brief description of the studies on populations living near incinerators. Click here for file

Additional file 3 **Studies on occupational exposures among incinerators and landfills workers**. The data provided represent a brief description of the studies on workers of waste management plants. Click here for file

Additional file 4 **Studies on other waste management processes**. The data provided represent a brief description of the studies on population living near plants using waste management technologies different from landfills and incinerators. Click here for file
